# MetaAnalysisOnline.com: Web-Based Tool for the Rapid Meta-Analysis of Clinical and Epidemiological Studies

**DOI:** 10.2196/64016

**Published:** 2025-03-06

**Authors:** János Tibor Fekete, Balázs Győrffy

**Affiliations:** 1 Department of Bioinformatics Semmelweis University Budapest Hungary; 2 Cancer Biomarker Research Group Institute of Molecular Life Sciences HUN-REN Research Centre for Natural Sciences Budapest Hungary; 3 Department of Biophysics Medical School University of Pecs Pecs Hungary

**Keywords:** statistics, pharmacology, treatment, epidemiology, fixed effect model, random effect model, hazard rate, response rate, clinical trial, funnel plot, z score plot

## Abstract

**Background:**

A meta-analysis is a quantitative, formal study design in epidemiology and clinical medicine that systematically integrates and quantitatively synthesizes findings from multiple independent studies. This approach not only enhances statistical power but also enables the exploration of effects across diverse populations and helps resolve controversies arising from conflicting studies.

**Objective:**

This study aims to develop and implement a user-friendly tool for conducting meta-analyses, addressing the need for an accessible platform that simplifies the complex statistical procedures required for evidence synthesis while maintaining methodological rigor.

**Methods:**

The platform available at MetaAnalysisOnline.com enables comprehensive meta-analyses through an intuitive web interface, requiring no programming expertise or command-line operations. The system accommodates diverse data types including binary (total and event numbers), continuous (mean and SD), and time-to-event data (hazard rates with CIs), while implementing both fixed-effect and random-effect models using established statistical approaches such as DerSimonian-Laird, Mantel-Haenszel, and inverse variance methods for effect size estimation and heterogeneity assessment.

**Results:**

In addition to statistical tests, graphical representations including the forest plot, the funnel plot, and the *z* score plot can be drawn. A forest plot is highly effective in illustrating heterogeneity and pooled results. The risk of publication bias can be revealed by a funnel plot. A *z* score plot provides a visual assessment of whether more research is needed to establish a reliable conclusion. All the discussed models and visualization options are integrated into the registration-free web-based portal. Leveraging MetaAnalysisOnline.com's capabilities, we examined treatment-related adverse events in patients with cancer receiving perioperative anti–PD-1 immunotherapy through a systematic review encompassing 10 studies with 8099 total participants. Meta-analysis revealed that anti–PD-1 therapy doubled the risk of adverse events (risk ratio 2.15, 95% CI 1.39-3.32), with significant between-study heterogeneity (*I*^2^=95%) and publication bias detected through the Egger test (*P*=.02). While these findings suggest increased toxicity associated with anti–PD-1 treatment, the *z* score analysis indicated that additional studies are needed for definitive conclusions.

**Conclusions:**

In summary, the web-based tool aims to bridge the void for clinical and life science researchers by offering a user-friendly alternative for the swift and reproducible meta-analysis of clinical and epidemiological trials.

## Introduction

Systematic literature reviews in medicine summarize the best-proven knowledge from a research domain into a single paper [1]. A meta-analysis is the statistical combination of results from at least 2 separate studies which can serve as a powerful tool for summarizing, integrating, and interpreting quantitative research findings from the collected sources. The first potential advantage of a meta-analysis is its capacity to enhance precision. Many studies, when considered in isolation, may be too small to provide convincing evidence about intervention effects, and a meta-analysis of multiple studies can provide the necessary power to investigate whether interventions have an impact on the incidence of rare events.

Second, meta-analyses allow for the exploration of questions not explicitly addressed by individual studies. Primary studies often focus on specific participant types and interventions with well-defined parameters. By incorporating studies with diverse characteristics, it becomes possible to investigate the consistency of effects across a broader range of populations and interventions. Additionally, this approach may shed light on reasons for variations in effect estimates.

Third, meta-analyses can play a role in settling controversies that may arise from apparently conflicting studies or in generating new hypotheses. The statistical synthesis of findings enables formal assessment of conflicting results, providing a means to explore and quantify the reasons for different outcomes.

On the other hand, meta-analyses can be limited by inadequate search strategies, lack of clarity on comparators, insufficient assessment of risk of bias in primary studies, failure to address heterogeneity across studies, and the use of inappropriate or nonstandard methodological approaches, all of which can undermine the reliability and validity of the conclusions drawn [2]. Recent systematic evaluation of meta-analyses focusing on digital biomarker interventions revealed significant methodological inadequacies, with 92% of studies demonstrating critically low quality, highlighting the need for more rigorous methodological approaches in meta-analytic research [3]. Furthermore, meta-analyses often fail to adequately consider important demographic factors and subgroup analyses, as evidenced by a recent overview of systematic reviews on digital technologies in chronic obstructive pulmonary disease where only 1 out of 30 reviews included age-based subgroup analysis, despite the potential impact of such factors on treatment outcomes and clinical applicability [4].

Here, the study aims to establish a new web portal for the simple and rapid execution of meta-analysis studies. In a meta-analysis, first, a summary statistic is computed for each study to describe the observed intervention effect uniformly across all studies. Then, a combined intervention effect estimate is calculated as a weighted average of the intervention effects estimated in the individual studies. We list options used by our web-based tool and for an all-inclusive description of the different approaches we suggest consulting statistical papers like the publications by Haidich [5] and by Tawfik et al [6]. In the last part of our paper, we describe a sample application where we analyzed the correlation between immune checkpoint blockade and adverse events.

## Methods

### Web-Based Meta-Analysis Portal

Our main goal was to create a web-based application that allows users to perform a complete meta-analysis of clinical trials with minimal effort. For this, we established a user-friendly portal that is accessible in any major web browser. The web application is running on an Ubuntu server (Canonical Ltd) powered by Apache. The application can be used to perform meta-analyses using binary (using total and event numbers), continuous (using mean and SD data), and time-to-event data (using hazard ratio and CI data). The results of each study can be pasted from commonly used spreadsheet applications like Microsoft Excel.

The calculations and plots used by the web-based system are done using *meta, RTSA,* and *metafor* packages in R programming environments (version 4.2.2; R Foundation for Statistical Computing). The Shiny user interface was created by using the *shinyjs*, *shinydashboard,* and *rhandsontable* R packages. The portal generates a forest plot to summarize the results of the meta-analysis. A 2-tailed *P* value below .05 indicates significance and is displayed in the analysis results. MetaAnalysisOnline.com uses the DerSimonian-Laird estimator to estimate tau-square [7].

In addition to the forest plot, a funnel plot is displayed to detect potential bias visually, and a *z* score plot is used for assessing the power achieved by the cumulative sample number. While there is commercial software available for performing a meta-analysis [8], the portal we developed can be accessed completely free, without the need for registration or logging in.

### The Implemented Statistical Methods

Experimental data are typically classified into 2 main categories: clinical data, which detail the effects of specific treatments on individual participants; and epidemiological data, which unveil patterns of disease or mortality in groups of participants exposed to either single agents or various substances. The most common classification in such studies uses categorical variables. A categorical variable is nonnumerical, relying on qualitative properties such as treatment arm, race, or gender, among others. Unlike numerical variables, categorical variables lack a specific ordering and assume values from a finite set of possibilities.

In MetaAnalysisOnline.com, the 2 study arms can be compared by either the fixed-effect model or the random-effect model. The fixed effect model operates on the assumption that a single true effect size underlies all studies in the meta-analysis, hence the term “fixed effect” or “common-effect” or sometimes “equal-effects.” Any variations in observed effects are attributed to sampling error. The random effects model posits that the true effect may vary across studies due to inherent differences or heterogeneity between them. As studies encompass diverse participant compositions and different interventions, individual studies may yield varying effect sizes. If an infinite number of studies were conducted, the effect estimates from all studies would conform to a normal distribution and the pooled estimate would then represent the mean or average effect. The assumption is that the effect sizes observed in conducted studies represent a random sample from the universe of all possible effect sizes, hence the term “random effects” [9]. The random effects approach for aggregating evidence from a series of experiments that compare 2 treatments was originally proposed by DerSimonian and Laird [7]. They integrated the heterogeneity of effects into the analysis, providing a comprehensive assessment of the overall treatment efficacy. The heterogeneity can be assessed using the *I*^2^ and 𝜏^2^ statistics, where the 𝜏^2^ represents the between-trial variance of the effect size. The square root of the 𝜏^2^ provides an estimate of the SD of the effects in the analyzed studies. High values of 𝜏^2^ indicate substantial heterogeneity among the effect sizes, suggesting that the true effects are not consistent across studies and may be influenced by other factors [10]. The *I*^2^ method, developed by Higgins and Thompson [11], offers a percentage value representing the variability observed in the effect size, independent of sampling error. One of its advantages lies in the ease of interpreting its results: *I*^2^ percentages of 25% correspond to low, 50% to medium, and 75% to high levels of heterogeneity, respectively [12].

There are 4 commonly used methods of meta-analysis for dichotomous outcomes: 4 fixed-effect methods (Mantel-Haenszel [13], Peto, Bakbergenuly [14], and inverse variance) and 1 random-effect method (DerSimonian and Laird inverse variance [7]). The Peto method is limited to combining odds ratios (ORs), while the other 3 methods can combine ORs, risk ratios, or risk differences. The Mantel-Haenszel method is a fixed effect meta-analysis method that uses a different weighting scheme depending on the type of effect measure being used (eg, risk ratio or OR) [13]. A relatively recent development is the Bakbergenuly sample-size-weighted estimator approach, which offers an alternative by using a weighted average where each study’s weight is determined by its effective sample size [14]. In the fixed effect model, the weights are calculated as w_i_=1/v_1_ where v_1_ represents the variance of the study. In the calculation, also known as the inverse variance method, studies with lower variances are weighted higher. In the random effect model, the calculation of weights is different, and the formula is 𝑤_i_=1/ (𝜏^2^+v_i_), where 𝜏^2^ represents the square of the variance of the distribution of true effect sizes. The inverse-variance method’s key advantage is its wide applicability, as it can be used for all effect measures and not only for OR, risk ratio, or risk difference. For its execution, we need to have the point estimate and the variance of the treatment effect in each study [7].

Quantitative variables can be either discrete or continuous. Discrete variables are constrained to a finite number of values (such as whole numbers), whereas continuous variables can assume any value, including all possible values within a given range (extending to an infinite number of decimal places). Standard methods for meta-analysis of continuous data rely on the assumption that the outcomes follow a normal distribution within each intervention arm in each study. The 3 commonly used summary statistics for a meta-analysis of continuous data include the mean difference (MD), the standardized mean difference (SMD), and the ratio of means. The choice of summary statistics for continuous data primarily depends on whether studies report the outcome using the same scale (in which case MD is used) or different scales (in which case the SMD is typically used). In the MD approach, the SDs and sample sizes are used to calculate the weight assigned to each study and studies with smaller SDs are given relatively larger weights. In the SMD approach, the SDs are used to standardize the MDs to a single scale and are also used in the computation of study weights. When comparing with MD, interpreting the results can be challenging due to the need to categorize the SMD. Typically, we use the categories outlined by Cohen, which indicate that an SMD of approximately 0.2 corresponds to a small effect, around 0.5 signifies a moderate effect, and approximately 0.8 indicates a large effect [15]. In addition to MD and SMD, MetaAnalysisOnline.com also includes the ratio of means, a more recent solution where instead of using the difference between groups, the log-transformed ratio of the means of the groups is used in the analysis. The advantage of this approach is that the output of the result is easily interpretable similar to the odds or risk ratio [16].

### Demonstration: The Correlation Between Immune Checkpoint Blockade and Adverse Events

In recent years, the addition of immune checkpoint blockade using anti–PD-1 drugs, such as pembrolizumab, nivolumab, and cemiplimab, into cancer therapy has improved clinical outcomes. However, the safety of the immune checkpoint blockade needs further evaluation. As a demonstration of the functionalities of MetaAnalysisOnline.com, a set of previous studies was analyzed to assess how incorporating immune checkpoint blockade into perioperative cancer therapy affects treatment-related adverse events in patients treated with anti–PD-1 therapy.

## Results

### Graphical Representation of the Overall Effect: The Forest Plot

A comprehensive forest plot displays a set of the following information.

- The name of the original studies used in the meta-analysis. It is crucial to ensure the uniqueness of study names, particularly when an author or study name is listed more than once.
- The number of events and the total number of participants in each group of the study.
- A graph representing the relative risk and 95% CIs. Each square in the plot embodies the study results, centered on the point estimate, with a horizontal line indicating the 95% CI. The diamond represents the overall meta-analysis estimate, with the center signifying the pooled estimate and the prediction interval indicating the confidence limits. The CI depicts the range of intervention effects compatible with the study’s result, while the size of the block draws attention to the studies with larger weight, which dominates the calculation of the summary result presented by the diamond at the bottom.
- The risk ratio or hazard ratio and 95% CI for each individual study and the overall meta-analysis.
- The percentage of weight assigned to each study, particularly when presenting pooled results. Studies with higher sample numbers or those with narrower CIs receive higher weights.
- Finally, at the bottom of the plot, the heterogeneity and the overall effect values. A significant *P* value for heterogeneity indicates a substantial difference between studies. However, they heavily rely on the assumption of a normal distribution for the effects across studies and can present challenges when the number of studies is small, potentially leading to artificially wide or narrow intervals.

**Figure 1:**
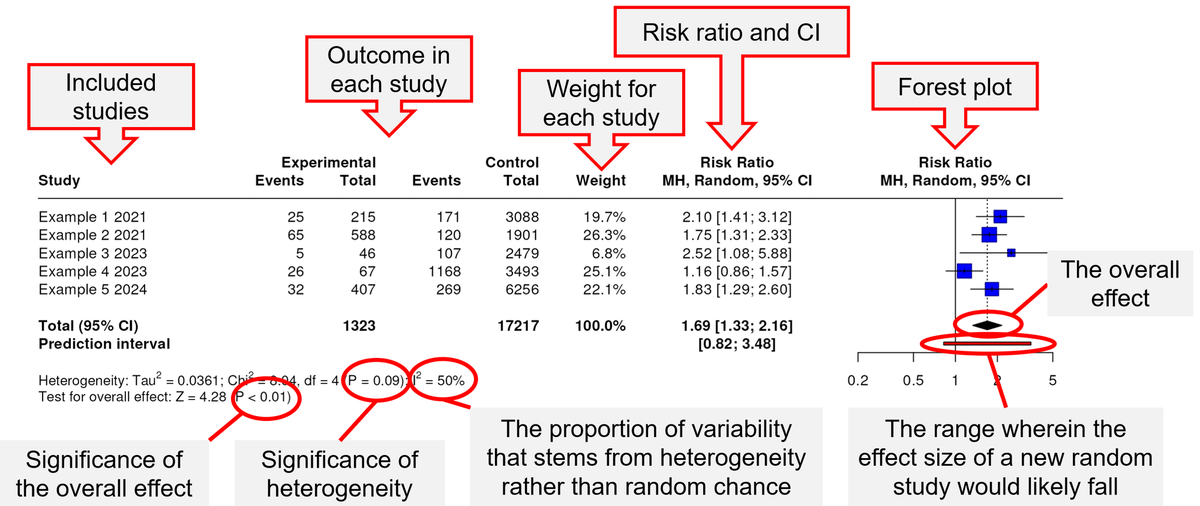
Components of a forest plot.

**Figure 2:**
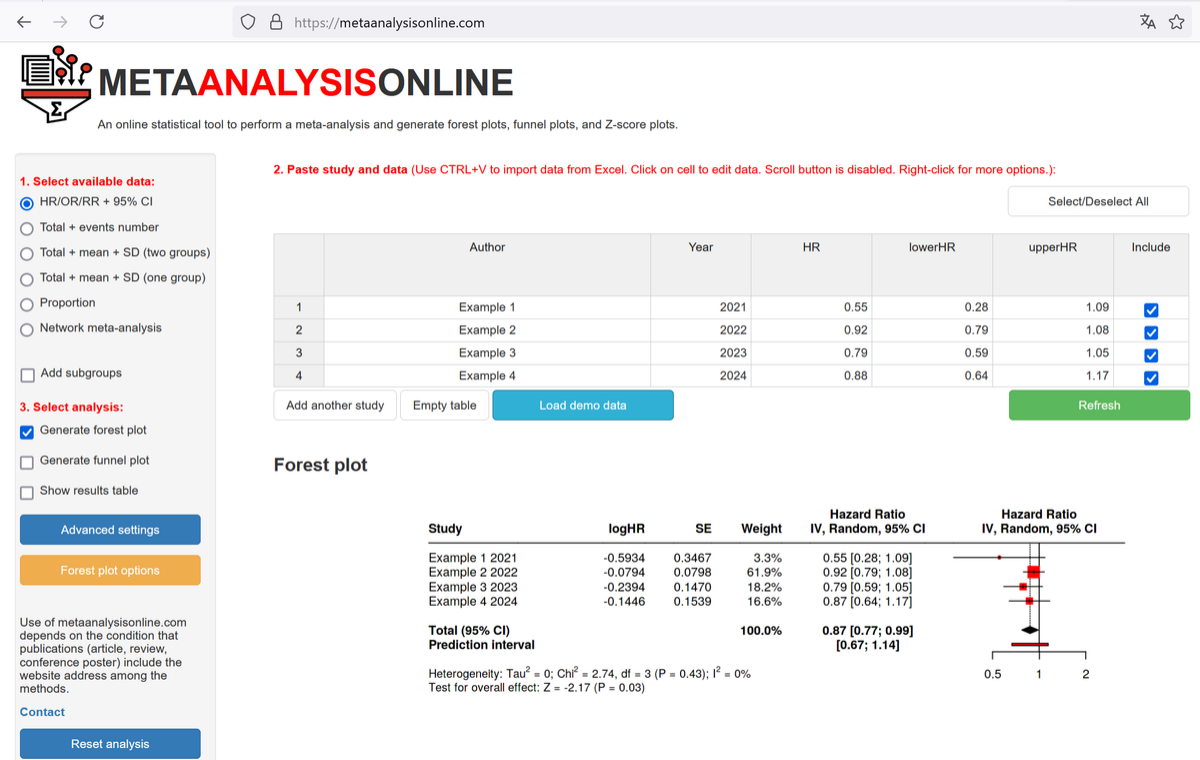
Screenshot of the web-based analysis interface showing the demonstration data and the generated forest plot.

The features discussed in this section are marked in the plot generated by demo data in Figure 1. In addition, a screenshot of the analysis page with a generated forest plot is shown in Figure 2.

### Graphical Representation of Heterogeneity: The Funnel Plot

**Figure 3:**
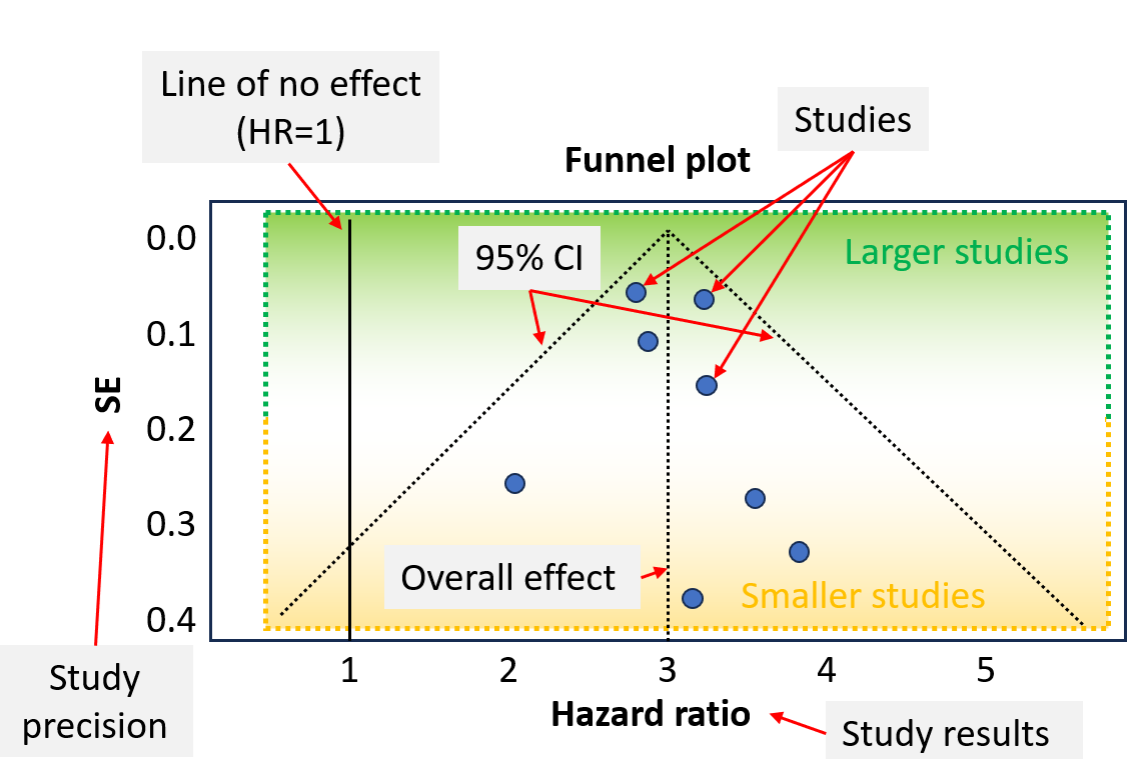
Components of a funnel plot. HR: hazard ratio.

The second analysis available in MetaAnalysisOnline.com is the funnel plot designed to detect publication bias. Whether the primary studies for pooling are observational studies or randomized controlled trials, heterogeneity between studies is probable. We show the components of a funnel plot used to assess study heterogeneity in Figure 3. Each dot on the plot represents an individual study. The x-axis represents the hazard ratio, or in other cases the log of OR, indicating the effect size of the treatment in each study. The y-axis represents the standard error, reflecting study precision, with larger studies having better precision (within the green area) and smaller studies having worse precision (within the orange area). In the plot, the vertical solid black or red line serves as the reference line corresponding to no effect or a hazard rate of 1 (or log OR=0). The overall effect and 95% CIs are depicted by the dashed black lines.

If the funnel plot suggests the presence of publication bias, the trim-and-fill method can be used to estimate the number of missing studies and impute their effect sizes. This allows the meta-analysis to be “trimmed” of asymmetric studies, and “filled” with the estimated missing studies to create a more symmetric funnel plot. The web-based platform automatically displays a button to generate trim-and-fill plots in case of a significant Egger test.

### Trial Sequential Analysis and the z Score Plot

**Figure 4:**
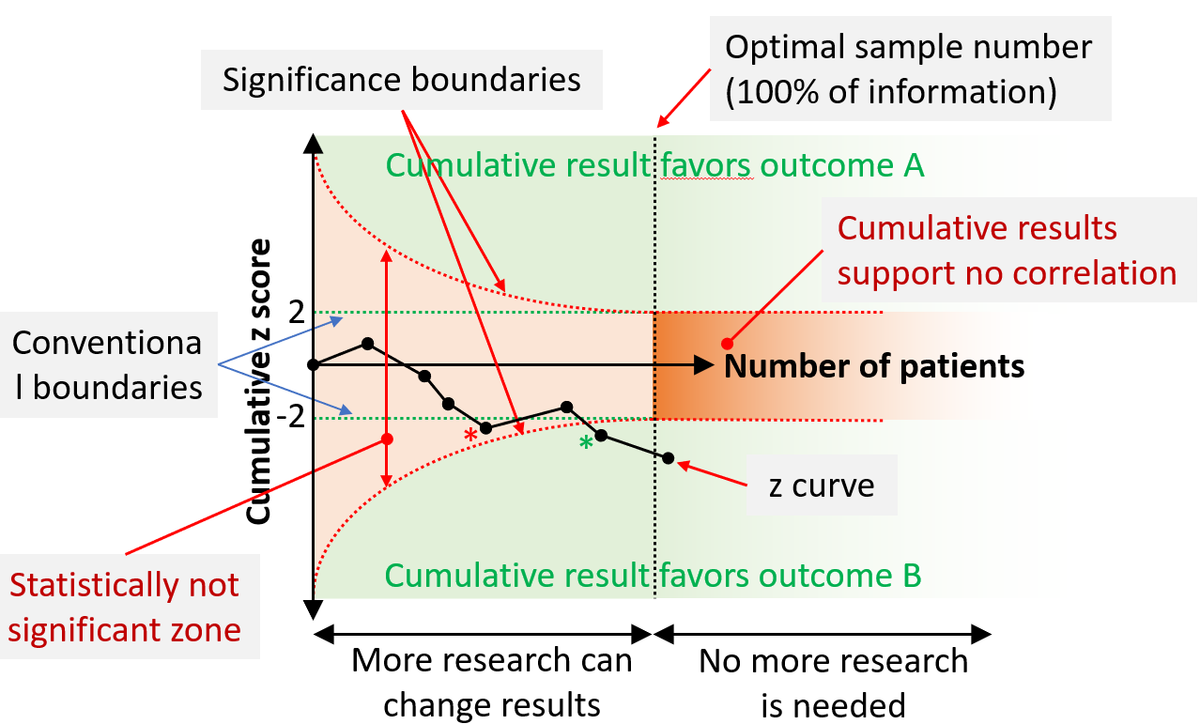
The z score plot visualizes the robustness of the sample number. The plot displays the correlation between the cumulative sample number and significance after the addition of each new study. Once the optimal sample number is reached, a definitive conclusion can be drawn about the investigated condition. As the displayed z curve includes more and more patients, it seems to favor outcome B by the conventional cut-offs—however, this sample number is not yet sufficient for statistical significance (red asterisk). As further studies are included, the cumulative outcome reaches statistical significance (green asterisk). Finally, after adding the last study, the z curve surpasses the optimal sample number indicating that no further research is needed.

MetaAnalysisOnline.com performs a trial sequential analysis by drawing the *z* score plot to illustrate the reliability of the sample size. The y-axis of a *z* score plot depicts the cumulative *z* score and the x-axis the cumulative sample size. The cumulative *z* curve is built by sequentially adding studies based on chronological order. The plot depicts the relationship between the collective sample size and the achieved significance. When the ideal sample size is attained, a decisive conclusion regarding the examined condition can be made. The components of a *z* score plot are visualized in Figure 4.

At the end of the *z* curve lies the most recently added study, falling into one of the following zones: “favoring outcome A,” “favoring outcome B,” “no correlation,” or “statistically not significant.” The first 2 zones indicate statistically significant results, which may vary until the optimal sample size is reached, but after this, a reliable final conclusion can be determined. Placement in the “no correlation” signifies strong evidence suggesting that subsequent studies are unlikely to alter the no-effect outcome significantly. Placement within the “not statistically significant” zone indicates the definitive requirement for further studies as neither correlation nor missing correlation could be determined.

### Independent Validation

To verify the output of the digital portal, a set of 10 samples was drawn from the dataset of a simulated randomized trial developed for the *metafor* package and a meta-analysis was performed. Analysis was repeated using IBM SPSS (version 29). The following outputs were compared: OR, the calculated weight, overall effect, as well as measures of heterogeneity (*I*^2^ and τ²). For all parameters, the corresponding 95% CIs were also checked. The output results of the 2 methods were identical, indicating sufficient reliability of the implemented procedures.

### Demonstration: Immune Checkpoint Blockade and Adverse Events

**Figure 5:**
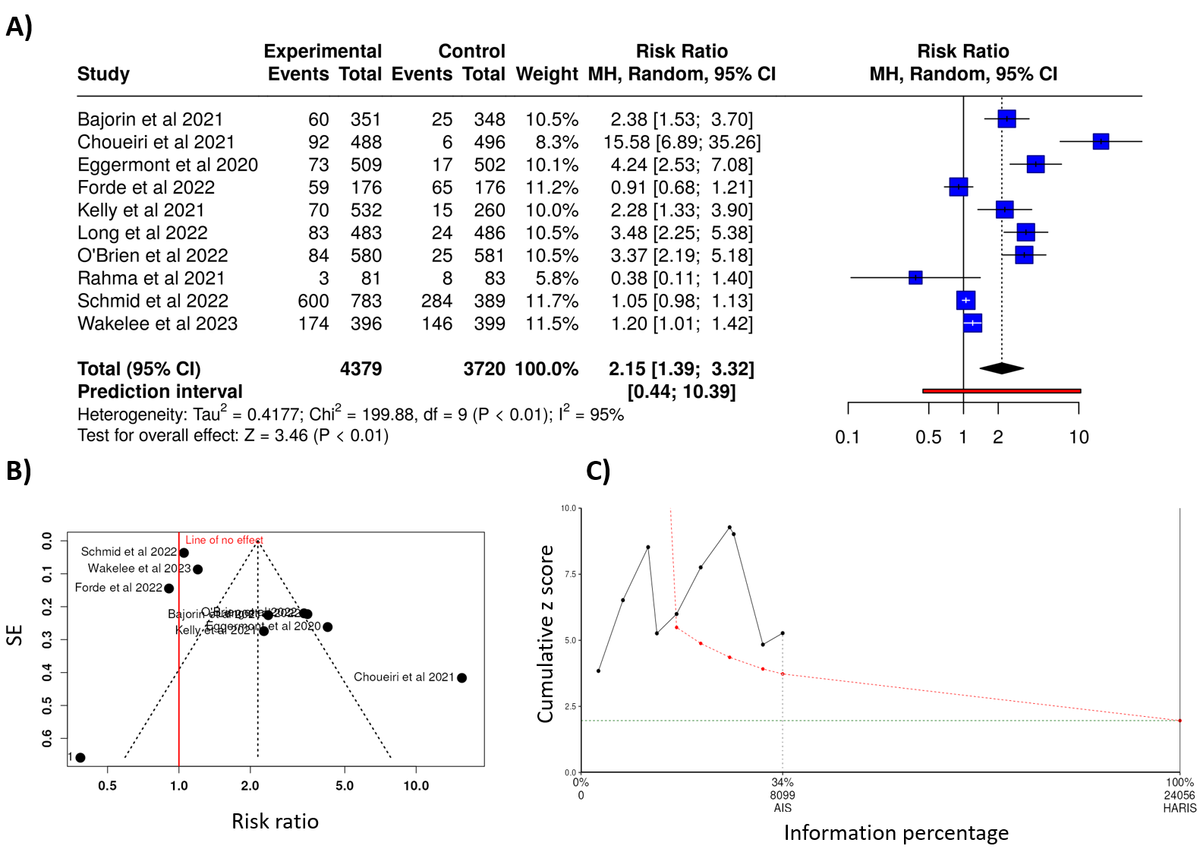
(A) Immune checkpoint blockade by anti-PD-1 drug class significantly increases adverse events. However, the funnel plot shows a (B) potential publication bias and (C) the z score plot indicates insufficient patient number of patients to draw a definitive conclusion (at significance=.05, type II error level=.2, and minimal clinically relevant outcome=30%) [30-39]. AIS: actual information size; HARIS: heterogeneity adjusted recommended information size; MH: Mantel-Haenszel method.

To show the entire analysis pipeline, the association between immune checkpoint blockade using anti–PD-1 agents and adverse events was analyzed. Altogether 10 studies were included with a total of 4379 participants in the anti–PD-1–treated cohort and 3720 participants in the control cohort [17-26]. Based on the analysis performed using the random effects model with the Mantel-Haenszel method to compare the risk ratio, there is a statistical difference between the 2 cohorts, the overall risk ratio is 2.15 with a 95% CI of 1.39-3.32. The test for overall effect reached significance as depicted in Figure 5A.

Notably, substantial heterogeneity was detected, suggesting inconsistent effects in magnitude and/or direction. The *I*^2^ value indicates that 95% of the variability among studies stems from heterogeneity rather than random chance.

The funnel plot indicates a potential publication bias. The Egger test supports the presence of funnel plot asymmetry (intercept: 3.85, 95% CI 1.39-6.3; *t*_8_=3.072; *P*=.02; visualized in Figure 5B).

Based on the *z* score plot, the total number of samples did not reach the optimal number, and more research is needed to establish a conclusion (Figure 5C).

## Discussion

Here, we have established a new aid for those performing systematic reviews and meta-analyses. A major advantage of our tool is its direct design for life sciences researchers and medical professionals who generally lack extensive statistical and bioinformatic skills. The user-friendly interface enables the execution of the analysis in real-time. Entering the data is possible by directly copying from a database with a single click. The tool generates the 3 most important graphical representations–a forest plot, a funnel plot, and a *z* score plot—in real-time. The most important advantage of the tool over other software lies in its extremely user-friendly setup—neither installation nor registration is necessary for the use and the input page was set up to enable prompt copying of data from popular spreadsheet applications.

The most important graphical result provided by MetaAnalysisOnline.com is a forest plot, which serves as a visual depiction of both the outcomes from individual studies and the overall results of the analysis [27]. Additionally, it illustrates overall effect estimates and the heterogeneity of studies, reflecting the variation in results among individual studies [28]. The plot also includes the prediction interval, which is widely used to express the amount of heterogeneity in a meta-analysis [29]. Notably, many reviews in the literature use slightly different forest plots as there is no uniform standardization on their appearance. A previous study investigating over 2000 forest plots from the literature identified highly standardized plots in Cochrane reviews but missing key elements in non-Cochrane reviews. Certain formats were not optimal for information exchange, and a significant number of plots contained insufficient data to be considered useful [30].

The second implemented visual result is the funnel plot. In this, results from smaller studies will scatter broadly at the bottom of the graph, while the dispersion is likely to decrease among larger studies. In the absence of bias, the plot will take on the appearance of a symmetrical, inverted funnel, with asymmetry suggesting the presence of a risk of publication bias [31]. A significant Egger test result means that the points on the funnel plot are not symmetric. It is important to acknowledge that in practical scenarios, the source of heterogeneity cannot be determined and is often unknown. It is important to distinguish between various types of heterogeneity. Clinical heterogeneity refers to variability in participants, interventions, and outcomes studied, while methodological heterogeneity pertains to variability in study design, outcome measurement tools, and risk of bias. Statistical heterogeneity arises from variability in the intervention effects being evaluated in different studies, which can be a result of clinical or methodological diversity, or both. *I*^2^ and 𝜏^2^ statistics can be used to assess study heterogeneity [32] and although the funnel plot is primarily used to detect publication bias, it can also be used to visualize the heterogeneity [33]. To provide a meaningful summary, it is crucial to ensure that the group of studies is sufficiently homogeneous in terms of participants, interventions, and outcomes before conducting a meta-analysis.

A fundamental question omitted in many performed meta-analyses is assessing the robustness of the sample number reached [34]. In particular, meta-analyses face the risk of yielding misleading outcomes due to significant results that may be false positives (type I errors; α) or falsely insignificant findings (type II errors; β). These errors can stem from various sources such as low-quality or underpowered trials, publication bias, and excessive significance testing. To address this issue, trial sequential analysis (TSA) has been devised as a cumulative meta-analysis technique [35]. TSA aims to account for both type I and type II errors, providing a means to estimate the point at which the observed effect size becomes robust enough to resist further influence from additional studies. In other words, TSA offers guidance on the necessity of further studies and helps clinicians prevent unnecessary trials [36].

Our tool was established to incorporate a simple way to conduct subgroup analysis as well—to examine the potential variability of treatment effects among different subgroups of patients or trials [37]. Subgroup analysis involves dividing all participant data in the meta-analysis into subsets based on patient characteristics (eg, age) or trial characteristics (eg, location), and then performing a meta-analysis on one or more of these subsets. These analyses can help to estimate treatment effects for clinically relevant subgroups of patients or to identify sources of heterogeneity [38]. The decision to conduct such analyses may be based on prior research suggesting that treatment effects could differ among different patient subgroups.

Two considerations should be kept in mind when running the statistical analysis using MetaAnalysisOnline.com. Sometimes the data points scatter over a wide range and truncating the data is advisable (to constrain the length of the follow-up time, the applied treatment dose, or any other descriptive characteristics of the participants) at a selected threshold in case of scarcity of data above the threshold value. A second issue refers to the inherent differences between the studies due to the varying number of included participants. To achieve the most robust results, studies should be weighed for sample size to better reflect the characteristics of the general population.

We demonstrate the use of MetaAnalysisOnline.com by performing a meta-analysis of studies examining the association between immune checkpoint blockade for anti-PD-1 agents and adverse events [39]. Although immune checkpoint blockade significantly increased the rate of adverse events, we observed a significant heterogeneity, and we can conclude that more research is needed to establish an accurate correlation.

Despite the meta-analysis’s aim to identify and evaluate all relevant studies meeting inclusion criteria, this goal may not always be fully realized. Some studies might be overlooked, particularly if they are not published in English or if they report nonsignificant results, making them less likely to be published. Understanding and accounting for these factors is essential for a comprehensive and critical appraisal of a meta-analysis.

Finally, we have to discuss some limitations of our tool. In order to retain user-friendliness, we had to make some compromises. The maximal number of studies in 1 analysis is limited to 100. We have established a default format for the plots, and the graphical parameters (eg, font type and size, spacing, and line thickness) cannot be changed. In addition, we used Shiny to enable seamless visualization and scaling. Because of this, the server behind the platform can sometimes slow down when a large number of studies are analyzed in 1 setting.

In summary, MetaAnlysisOnline.com can be used for a quick and comprehensive meta-analysis in clinical and epidemiological trials. When presenting the results of a meta-analysis, 3 key aspects need to be considered. First, we have to examine the pooled result, representing the overall combined outcome obtained by pooling the individual studies in a forest plot. While providing a synthesized perspective on the collective findings, we have to consider heterogeneity, which relates to variations in results, methodology, or study populations across the included studies. Second, we must assess the potential presence of the risk of publication bias. Finally, the *z* score plot can be used to determine whether the cumulative sample number is sufficient for a definitive conclusion, or whether additional studies are still needed.

## Abbreviations

MD: mean difference
OR: odds ratio
SMD: standardized mean difference
TSA: trial sequential analysis

## Data Availability

All data generated or analyzed during this study are included in this published article.
